# Anomalous Aortic Origin of a Coronary Artery in Pediatric Patients

**DOI:** 10.1007/s40124-024-00317-7

**Published:** 2024-05-24

**Authors:** Tam T. Doan, Charles Puelz, Craig Rusin, Silvana Molossi

**Affiliations:** 1 Coronary Artery Anomalies Program, Division of Cardiology, Texas Children’s Hospital, 6651 Main Street MC-E1920, Houston, TX 77030 USA; 2 Department of Pediatrics, Baylor College of Medicine, Houston, TX 77030 USA

**Keywords:** Anomalous aortic origin of a coronary artery, Sudden cardiac death, Sudden cardiac arrest, Inducible myocardial ischemia, Computational modeling, Children

## Abstract

**Purpose of Review:**

We present a contemporary approach to risk assessment and management of patients with anomalous aortic origin of a coronary artery (AAOCA).

**Recent Findings:**

Anomalous left coronary artery from the right aortic sinus (L-AAOCA) with interarterial course carries a high risk of sudden cardiac death (SCD); therefore, current guidelines recommend exercise restriction and surgical intervention. Recent data in intraseptal and juxtacommissural L-AAOCA showed inducible perfusion abnormalities, leading to consideration of surgical intervention. Anomalous right coronary artery from the left aortic sinus (R-AAOCA) carries a much lower risk and stress perfusion imaging is helpful in identifying patients with inducible ischemia. Perfusion abnormalities resolve following successful surgical intervention of AAOCA. Computational modeling techniques identifying risk features shows promise in the evaluation of AAOCA.

**Summary:**

Stress perfusion imaging is helpful in assessing AAOCA upon presentation and following surgical intervention. Computational modeling has potential in bridging knowledge gaps in AAOCA.

**Supplementary Information:**

The online version contains supplementary material available at 10.1007/s40124-024-00317-7.

## Introduction

Congenital anomalies of the coronary arteries represent a varied group of lesions and their embryological development is not completely understood [1, 2]. It may occur as an isolated anomaly or in association with other congenital heart diseases. While many coronary artery anomalies are detected as incidental findings with little to no significant consequence, approximately 20% may have a potential risk of coronary ischemia leading to myocardial infarction, arrhythmia, sudden cardiac arrest (SCA), and sudden cardiac death (SCD) [1, 3].

The true prevalence of AAOCA remains unknown as studies have focused primarily on symptomatic patients and individuals with cardiac imaging studies. The estimated frequency of left (L)-AAOCA is 0.03–0.15% while that of right (R)-AAOCA is 0.28–0.92% [1, 2]. AAOCA is reported to be one of the leading causes of SCD in young athletes, estimated 15–20% in this population [3, 4]. The risk of SCD is highest in young individuals during or immediately after intense exertion, particularly more in those with interarterial/intramural L-AAOCA but also reported in R-AAOCA [5–9]. Studies in adults with R-AAOCA undergoing conservative therapy report a mortality < 1% on follow-up at 5 years [2, 10].

This report focuses on the anatomy, physiology, diagnostic strategy and contemporary management of AAOCA. Additionally, we review recent advancement using computational modeling techniques and explore future directions aimed at bridging knowledge gaps in AAOCA.

## Anatomic Subtypes and Pathophysiology

Anatomic subtypes reviewed include R-AAOCA (most common), L-AAOCA with interarterial/intramural (Supplemental Fig. 1), intraseptal L-AAOCA, and rarely more posteriorly near the commissure between the left-coronary and non-coronary sinus (juxtacommissural L-AAOCA) [1, 11•, 12••, 13••, 14, 15••, 16]. Anatomic features considered as high-risk include interarterial course, intramural course, high ostial location, acute takeoff angle, and slit-like ostium, which may lead to SCD in the young [4, 15••, 17, 18]. Patients with R-AAOCA typically demonstrate these high-risk features regardless of whether patients exhibit exertional symptoms or inducible ischemia (Table 1) on stress perfusion imaging (SPI). On the contrary, patients with interarterial/intramural L-AAOCA do not always exhibit these features [12••, 19].

**Table 1:** Characteristics of R-AAOCA patients with (1) no/non-exertional symptoms versus those with exertional syncope and/or chest pain and (2) without versus with inducible ischemia (Texas Children’s Hospital 2012–2020) [12••]

(1) R-AAOCA and no/non-exertional symptoms versus those with exertional syncope and/or chest pain				
Parameter, n (%)	No or non-exertional symptoms(N = 168,76%)	Exertional symptoms(N = 52,24%)	Total(N = 220)	P value
Age at diagnosis, years (IQR)	9.2(4.5–13.7)	13.5(10.6–15.3)	11.4(6.1–14.5)	** < 0.001**
Males	103(61%)	30(59%)	133(60%)	0.79
CTA performed	137(82%)	52(100%)	189(86%)	
* Intramural course*	118(86%)	40(80%)	158(84%)	0.31
*Intramural length, mm (IQR) *	5(4–7)	5(4–7)	5(4–7)	0.65
*Stenotic/slit-like ostium *	99/134(74%)	38/50(76%)	137/184(75%)	0.77
*Acute angled takeoff *	136(99%)	51(100%)	187(99%)	0.54
EST performed	114(68%)	50(98%)	164(75%)	
* Positive*	2(1.7%)	0	2(1.2%)	0.52
SPI performed	120(71%)	49(96%)	169(77%)	
* Positive*	11(9%)	9(18%)	20(12%)	0.09
(2) R-AAOCA without inducible ischemia versus with inducible ischemia on stress perfusion imaging test				
Parameter, n (%)	No inducible ischemia(N = 149,88%)	Inducible ischemia(N = 20,12%)	Total(N = 169)	P value
Age at SPI, years (IQR)	13(10–15)	14(10–16)	13(10–15)	0.23
Males	93(62%)	12(60%)	105(62%)	0.83
Exertional symptoms	40(27%)	9(45%)	49(29%)	0.09
*Exertional chest pain *	37(24%)	8(40%)	45(27%)	0.15
*Exertional syncope *	10(7%)	1(5%)	11(7%)	1
CTA performed	147(99%)	20(100%)	167(99%)	1
*Intramural course *	126/146(86%)	19/20(95%)	145/166(87%)	0.47
*Intramural length, mm (IQR) *	5(4–7)	6.0(4.8–7)	5(4–7)	0.22
*Slit-like/stenotic ostium *	109/144(76%)	18/20(90%)	127/164(77%)	0.25
EST performed	135(91%)	20(100%)	155(92%)	0.38
*Positive *	0	2(10%)	2(1%)	**0.02**

*CTA* computerized tomographic angiography, *EST* exercise stress test, *SPI* stress perfusion imaging

Autopsy findings in those with SCD and AAOCA demonstrated diffuse myocyte necrosis, neutrophilic infiltrates and patchy replacement-type fibrosis [4]. Compression of the anomalous coronary artery and ostial abnormalities are postulated to cause myocardial ischemia and ventricular arrhythmia during exercise leading to SCA/SCD [4, 10, 17]. Recent invasive assessment of intracoronary flow indicates a two-tier pathophysiology: (1) Fixed obstruction from reduced cross-sectional area (slit-like ostium, proximal narrowing) and (2) Dynamic obstruction from lateral compression (acute take-off angle, intramural course, and elliptic vessel shape) [20•, 21, 22••].

## Clinical Evaluation

### Clinical Presentation and Initial Evaluation

In an autopsy report of 27 individuals with SCD from AAOCA, only 10 reported symptoms prior to the event: 4 with non-exertional symptoms and 6 with exertional syncope or chest pain [4]. Clinical studies reported patients with AAOCA may present with exertional chest pain, exertional syncope, or SCA, but 50% or more were asymptomatic or had non-specific symptoms at diagnosis [3, 11•, 12••, 13••]. Children were increasingly diagnosed through routine screening echocardiogram for a murmur, abnormal ECG, or family history of cardiovascular disease, as physical examination and baseline ECG in patients with AAOCA do not typically reveal specific findings [11•, 12••, 13••, 23, 24, 25•].

**Fig. 1:**
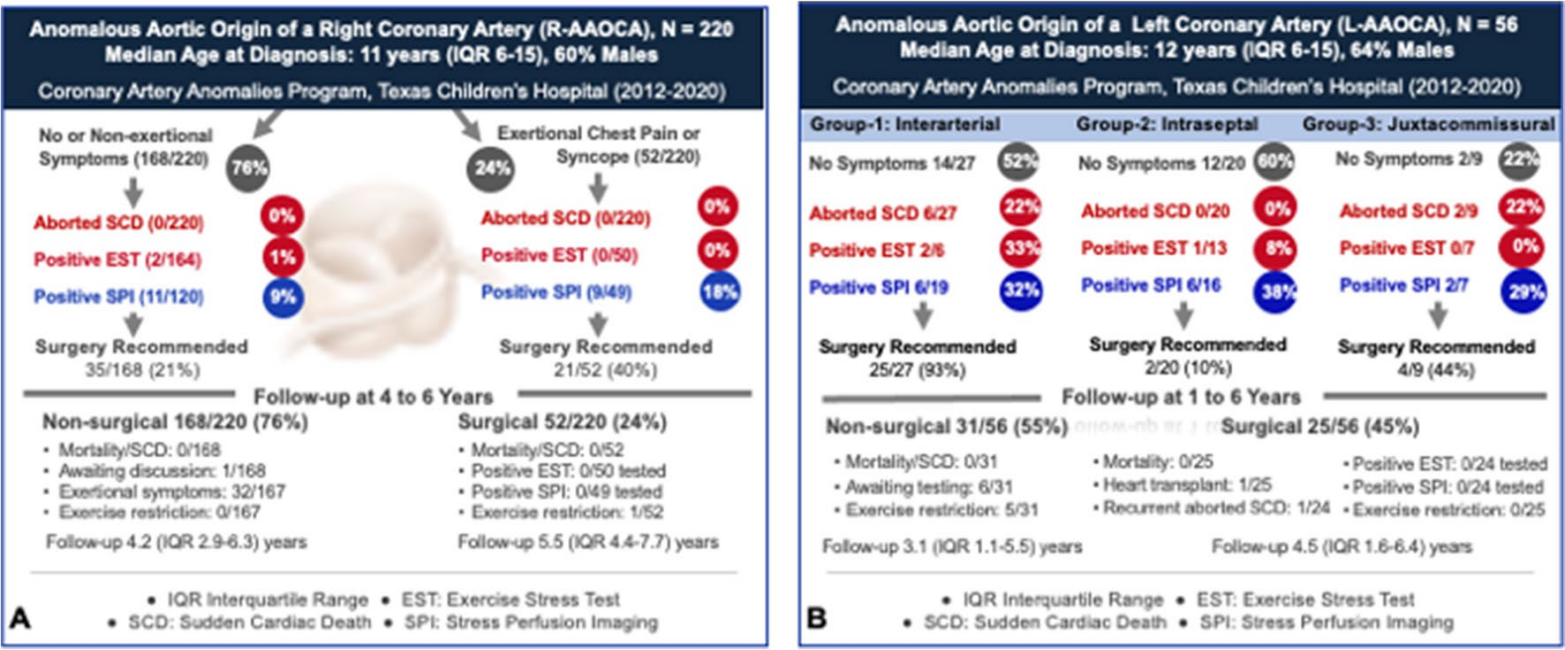
Clinical Characteristics, Risk Assessment, Management, and Contemporary Outcome of Anomalous Aortic Origin of a Right (A) and Left (B) Coronary Artery at Texas Children’s Hospital (2012–2020) [12••, 13••]^13^••

In 2012, Texas Children’s Hospital established the Coronary Artery Anomalies Program (CAAP) to standardize the assessment and management of children with AAOCA (Supplemental Fig. 2 & 3) [11•, 24]. From 2012 to 2020, the group reported 220 pediatric patients with R-AAOCA and 56 with 3 L-AAOCA subtypes (Fig. 1) [12••, 13••, 24]. While none of the patients with R-AAOCA or intraseptal L-AAOCA had SCA/SCD, 22% of patients with interarterial L-AAOCA presented with SCA and 22% of juxtacommissural L-AAOCA presented with SCA or cardiogenic shock (Table 2) [12••, 13••, 24, 26]. Exertional symptoms (including SCA in L-AAOCA) are more commonly seen in L-AAOCA (48% in interarterial, 40% in intraseptal, and 78% in juxtacommissural L-AAOCA) compared to 26% in R-AAOCA.

**Table 2:** Characteristics of patients with interarterial and juxtacommissural L-AAOCA presenting with sudden cardiac arrest or cardiogenic shock (Texas Children’s Hospital 2012–2020)

Patient	Age in Years	Prior Sports Participation	Circumstance of SCA	Symptoms prior to SCA	Initial ECG findings	AAOCA Anatomy	Surgery
1	12	Basketball	Basketball game	Exertional chest pain, dyspnea	Normal	Interarterial L-AAOCA, slit-like ostium, 2.5 mm intramural course	• 1st SCA: Unroofing* • 2nd SCA: LMCA translocation and LAD unroofing
2	14	Basketball	Basketball Practice	None	Nonspecific intraventricular conduction delay	Interarterial L-AAOCA, slit-like ostium, 3 mm intramural course	Unroofing
3	15	Football	Football Game	Non-exertional chest pain	Nonspecific intraventricular conduction delay	Interarterial L-AAOCA, elliptical ostium, 8.9 mm intramural course	Unroofing
4	15	Basketball	Basketball practice	None	Probable idioventricular rhythm, multiform ventricular ectopies	Interarterial L-AAOCA, slit-like ostium, 5 mm intramural course	Reimplantation, subsequent heart transplantation
5	16	Track	Non-competitive running	Pre-syncope	Normal	Interarterial L-AAOCA, elliptical ostium, 6.5 mm intramural course	Unroofing
6	18	Football	Football Practice	Palpitations, dyspnea	Normal	Interarterial L-AAOCA, slit-like ostium, 8.7 mm intramural course	Unroofing
7	0.4	None	None	None	Left axis deviation, Q waves (I and avL), T wave inversion in lateral leads	Juxtacommissural L-AAOCA, slit-like ostium, short intramural course	Unroofing
8	0.2	None	Cardiogenic shock	Shock	Biventricular hypertrophy, diffuse ST depression	Juxtacommissural L-AAOCA, slit-like ostium, short intramural course	Unroofing

*LAD* left anterior descending, *LMCA* left main coronary artery, *RCA* right coronary artery, *SCA* sudden cardiac arrest

^*^ Patient was cleared to return to full activities, presented with recurrent SCA [26]

Transthoracic echocardiography is the first-line imaging modality, able to identify interarterial and suggest intramural course but may not delineate coronary ostial morphology [12••, 13••, 16, 27]. Most experts recommend computed tomography angiography (CTA) or cardiac magnetic resonance imaging (CMR), with CTA offering superior spatial resolution, for reliable definition of the anatomy including ostial morphology, high ostial location, interarterial, intramural, or intramyocardial course [11•, 14, 15••, 16, 28•, 29, 30].

Most of the subjects in Basso’s report were asymptomatic prior to SCD, underscoring the challenge of evaluating asymptomatic young athletes with new AAOCA diagnosis [4]. Insights from studies utilizing CMR revealed evidence of delayed enhancement in middle-aged adults with R-AAOCA, but no significant scarring in children, challenging the perception of low risk in asymptomatic patients with R-AAOCA. The optimal method for assessing the risk of SCA in young individuals with AAOCA remains uncertain. While SPI has shown the highest yield in detecting an inducible myocardial ischemia, which is often considered as a surrogate indicator of SCA, the association between SPI findings and occurrence of SCA has not been definitively confirmed [16, 17, 30, 31••]. It is important to note that 9% of patients with incidental finding of R-AAOCA and 18% of R-AAOCA with exertional symptoms had an inducible myocardial ischemia demonstrated on SPI [32•]. Invasive assessment using resting instantaneous wave-free ratio (iFR), Dobutamine fractional flow reserve (FFR), Dobutamine iFR, and intravascular ultrasound (IVUS) provide helpful insights for the management in select cases of AAOCA [20•, 23, 24, 33].

### Non-invasive Stress Testing

#### Maximal Exercise Stress Test (EST)

EST is used in children who can tolerate exercise, although it has a low sensitivity to detect inducible ischemia in AAOCA [4, 12••, 24, 30, 34•, 35–37]. Utility of a maximal EST is challenged by variation in defining abnormal results, including blunted blood pressure response, premature ventricular contractions, and ST segment depression/elevation. Specific ST changes are regarded as having high specificity for myocardial ischemia [24, 36–38]. According to current consensus guidelines, asymptomatic patients with R-AAOCA are generally regarded as low-risk if the maximal EST results are normal [30, 37]. In a large series of 214 pediatric patients with R-AAOCA and EST, the occurrence of ischemic ST changes was very low (< 1%), while it was 11.5% (3/26) in patients with L-AAOCA [12••, 13••]. Despite its limited sensitivity, a maximal EST is valuable when there is specific ST changes consistent with myocardial ischemia.

#### Regional Wall Motion Abnormality (RWMA) by Stress Echocardiography or Stress CMR

Stress echocardiography can detect new RWMA or valvular dysfunction suggestive of ischemia following exercise or pharmacologic stress [39–43]. Rapid drop in heart rate post-exercise challenges accurate image acquisition and interpretation in children, whereas, pharmacologic stressors sustain peak heart rate, facilitating optimal image acquisition even in small children [44]. Stress echocardiography is cost-effective and accessible compared to other advanced imaging techniques. Despite challenges in RWMA assessment proficiency, it is used in various pediatric populations with abnormal coronary arteries [45]. Although not specifically reported in pediatric patients, stress echocardiography is preferred for evaluating inducible RWMA in some centers for children with AAOCA [36, 38, 44, 46]. Dobutamine, closely mimicking exercise by increasing contractility, decreasing systemic vascular resistance, as well as promoting coronary vasodilation, has been instrumental in the assessment of RWMA during maximal myocardial oxygen demand [47, 48]. In 51 pediatric patients with R-AAOCA, Dobutamine stress CMR (DSCMR) showed no notable difference in RWMA between patients with exertional symptoms and those with no/non-exertional symptoms [49•]. Despite being used in the assessment of patients with AAOCA, the yield of abnormal results are very low suggesting it may not be a sensitive test.

#### Stress Perfusion Imaging Testing

##### Nuclear Perfusion Imaging (NPI)

NPI with provocative stress is well-established for the evaluation of ischemic heart disease in adults, and its application in patients with AAOCA has been reported by different groups [24, 38, 46, 50, 51]. However, issues related to patient exposure to ionizing radiation and low spatial resolution leading to false positives and false negatives, resulted in decreased interest in its use in this population. DSCMR with first pass perfusion assessment has emerged as a safe, feasible, and reproducible alternative evaluating pediatric patients with AAOCA [24, 31••, 35, 52•].

##### DSCMR with First Pass Perfusion Assessment

In addition to assessing RWMA, first-pass perfusion enhances the sensitivity of DSCMR, aligning with the demand ischemia cascade where impaired perfusion precedes wall motion abnormalities [47, 48, 53, 54]. Doan and colleagues reported on 182 children with AAOCA undergoing 224 DSCMR studies at a median age of 14 years, and inducible perfusion defects were detected in 14% [31••]. Most studies were successfully completed without sedation, none with major events, and 12.5% experiencing minor events. This study demonstrated the safety and feasibility of DSCMR in pediatric patients with AAOCA, significantly impacting management decisions [31••]. Moreover, agreement between DSCMR and invasive FFR during Dobutamine challenge was demonstrated in 13 young patients with AAOCA [55]. Comparable data were observed in isolated case reports and intraseptal L-AAOCA [23, 56, 57]. Given these recent data, DSCMR clearly plays a pivotal role in detecting perfusion abnormalities in AAOCA, facilitating comparison of results before and after surgical repair, where applicable, to assess resolution of inducible ischemia [24, 31••, 35, 52•]. However, ensuring excellent image quality and expertise are important for the visual assessment of first-pass perfusion of gadolinium, specifically to distinguish dark rim artifacts from true inducible perfusion defects. Risk stratification in AAOCA remains challenging, although DSCMR undoubtedly aids in the management decision-making of these patients.

### Invasive Testing Under Provocative Stress

In recent years, invasive assessment of coronary artery flow has been performed when there is conflicting data between patient symptoms and non-invasive test results [21, 58]. By utilizing pharmacologic stressors to replicate physiological changes during exercise, this approach may reveal hemodynamically significant lesions warranting intervention. It involves angiographic assessment of the vessel diameter and measuring intracoronary hemodynamics using iFR and FFR [20•, 21, 23, 28•, 33, 56, 58, 59••, 60].

#### Fractional Flow Reserve (FFR)

FFR is a pressure-derived index of severity in coronary artery stenosis, calculated as a ratio of mean intracoronary pressure distal to the lesion (Pd) divided by the mean aortic pressure (Pa) for the entire cardiac cycle. It requires the use of a coronary vasodilator to unmask a fixed obstructive coronary lesion. In adults with ischemic heart disease, coronary revascularization is considered when FFR < 0.8. In cases involving potential dynamic coronary compression, as seen in AAOCA with intramural or intraseptal course, Dobutamine is favored as a pharmacologic agent to induce provocative stress mimicking physiological changes during exercise [31••]. Dobutamine has positive inotropy and increased cardiac output while decreasing systemic and coronary vascular resistance [61, 62]. Considering the potential overshoot of distal systolic pressure (Pd) leading to falsely normal FFR, diastolic FFR (dFFR) may be better than FFR in revealing coronary flow impairment during Dobutamine infusion [63]. Initial feasibility and safety on the use of FFR in children with AAOCA was reported by Agrawal et al. in 2017 in four patients with AAOCA, highlighting its role in risk stratification for select patients [58]. However, the manual calculation of dFFR (averaging three Pd/Pa ratio using digital calipers at end diastole) presents a major limitation.

#### Instantaneous Wave-Free Ratio (iFR)

iFR is a drug-free pressure-derived index of coronary artery flow during a period of naturally constant and low resistance, characterized by minimally competing pressure waves in diastole [64]. It does not require a vasodilator to reduce coronary vascular resistance and has shorter procedure time, leading to better patient tolerance [65]. iFR demonstrates better agreement with coronary flow reserve [66] and non-inferior to FFR as it relates to health outcomes when guiding coronary revascularization in ischemic heart disease [65–67]. Doan et al. reported the use of iFR in children with AAOCA and demonstrated that iFR correlated with adenosine FFR and Dobutamine dFFR, making it an alternative for patients in whom pharmacologic stressors like Dobutamine are contraindicated [33]. Additional data from the same group demonstrated the use of resting iFR and dFFR with Dobutamine challenge to guide decision-making in patients with concerning clinical symptoms but negative non-invasive perfusion studies under provocative stress [68•]. Abnormal values of intracoronary flow observed in these patients were shown to significantly improve or completely resolve on repeat invasive studies following surgical intervention [23, 28•, 60, 69]. However, it is important to note that iFR and FFR cutoff values are based on ischemic coronary artery disease in adults and may not be optimal in AAOCA [11•, 33, 60].

#### Intravascular Ultrasound (IVUS)

IVUS has been utilized in adults with AAOCA and considered the gold standard by some experts for assessing the intramural segment due to its excellent spatial resolution and ability to evaluate dynamic lateral compression both at rest and during pharmacologic stress [2, 18, 70, 71]. In adult patients with R-AAOCA, Angelini and colleagues used IVUS and demonstrated the most severe stenosis was in the proximal intramural segment, just distal to its ostium [70]. IVUS measured the diameter (minimal and maximal) of the anomalous coronary in the compromised area during systole and diastole and significant compression was considered when area ratio > 50% at baseline and/or > 60% during provocative stress [70]. IVUS has also guided stent placement in the proximal intramural segment in select adults patients with R-AAOCA [70]. Our team reported the utility of IVUS in pediatric patients with intraseptal AAOCA and myocardial bridges and its important roles in the management decision -making and postoperative assessment [23, 28•, 58].

While promising in a select group of patients with AAOCA, further data are needed to determine FFR, iFR, and IVUS role in risk stratification in young patients. Importantly, FFR, iFR, and IVUS should not be considered a common technique for evaluating young patients with AAOCA, as expertise is crucial to mitigate potential serious coronary complications associated with the procedure.

## Management Decision-Making

### Medical Management and Activity Restriction

Clinical follow up without medication or intervention is generally considered when the provocative testing, including SPI, shows no ischemic changes in the asymptomatic patients with R-AAOCA [12••, 24, 30, 72]. In general, surgical intervention is favored in pediatric patients with AAOCA and evidence of myocardial ischemia, when the benefits deemed to outweigh the risks. Beta-blocker therapy has been shown to mitigate symptoms in patients with intraseptal L-AAOCA. However, concerns about its negative impact on athletic performance exist and beta-blocker should only be considered for the youngest age group [56].

Exercise restriction is currently recommended for patients with interarterial L-AAOCA, regardless of symptoms, or those with other types of AAOCA who present with SCA, have evidence of inducible ischemia on provocative testing, or present with concerning exertional symptoms [13••, 24, 30, 37, 72]. The asymptomatic patient with R-AAOCA and no evidence of ischemia on provocative testing can participate in competitive sports/exercise following appropriate shared decision-making. Patients and families should be counseled about the rare risk of SCD and the unknown negative predictive value of a negative stress test [37]. It is also important to discuss and recommend preparedness for SCA with an emergency action plan, including CPR training and availability of an automated external defibrillator (AED).

Following surgical repair of AAOCA, we empirically prescribe antiplatelet therapy with aspirin for 3 months. Aspirin is discontinued after reassuring post-operative cardiac testing, including repeating all pre-operative testing. Athletes should consider sports participation without restriction if symptom-free, reassuring post-operative testing, and with resolution of abnormal findings seen preoperatively [24, 30, 35]. In patients who presented with SCA, a longer postoperative period up to twelve months might be necessary to ensure freedom of symptoms, arrhythmia, and no evidence of myocardial ischemia on provocative testing [30]. Return to play may be considered after negative postoperative testing at 3 months if asymptomatic and exercising shared decision-making [11•, 13••]. It is good practice to discuss emergency action plan including the availability of AED and trained personnel capable of performing cardiopulmonary resuscitation and using an AED [30].

### Surgical Approach

To date, the exact mechanisms of ischemia leading to SCA in AAOCA remains undefined [11•, 73, 74]. Surgical repair of AAOCA has been performed to potentially address this risk and mitigate the occurrence of SCA, although surgical indications and techniques remain with significant practice variation [35, 36, 52•, 75–77]. Consensus guidelines has provided a standardized approach recommending surgical intervention for those with signs and/or symptoms of ischemia [30, 37, 72]. In patients with reassuring diagnostic testing results, surgery is recommended for patients with R-AAOCA who had ventricular arrhythmia and all patients with interarterial/intramural L-AAOCA [72]. Patients who were diagnosed with R-AAOCA can be considered for surgery despite reassuring testing and no other clinical concern [72].

The goals of AAOCA repair were to yield an unobstructed coronary artery from the appropriate aortic sinus while minimizing the risk of procedural complications [11•, 32•, 35]. Surgical repair of AAOCA should aim at eliminating the intramural course and its associated ostial narrowing by unroofing, ostioplasty, or transection and reimplantation (TAR) [12••, 19, 30]. Unroofing of an intramural course is most commonly reported, although other techniques including TAR or neo-ostium creation have also been performed [35, 36, 52•, 75, 78, 79]. Surgical complication and reoperation due to coronary artery stenosis have been reported up to 5% at 7 years following the index operation from the multicenter Congenital Heart Surgeons Society Registry [75].

#### AAOCA with Interarterial Course or L-AAOCA from the Non-Coronary Sinus (juxtacommissural L-AAOCA)

At our center, the primary surgical strategies included unroofing of an intramural course and coronary TAR (Supplemental Fig. 4) [12••, 13••, 32•, 35, 52•]. Takedown of the aortic commissure in surgical unroofing is avoided due to the potential risk of postoperative aortic insufficiency [35]. Unroofing is preferred in patients with an intramural segment above the aortic valve commissure. It is a widely adopted technique and considered relatively safe in the surgical repair of AAOCA [36, 79, 80]. When the unroofing technique posed a risk of compromising aortic valve integrity, coronary TAR is performed in both adults and children [52•, 76, 81]. TAR is favored in patients with a short intramural length and the intramural segment traveling below the level of the aortic valve commissure, where surgical unroofing would not correctly position the ostium in its correct aortic sinus (Supplemental Fig. 5) [35, 52•]. It involves extensive manipulation of the anomalous coronary artery, including transection followed by reimplantation without utilizing an aortic button [52•]. It is important to note that the superiority of one surgical technique over the other remains unknown, and that TAR should only be considered for select candidates and performed in centers with specialized expertise due to its technical complexity with potential iatrogenic complications [82].

#### L-AAOCA with Intraseptal Course

Surgical options for this anomaly are limited, evolved over time, and the long-term outcomes are uncertain. Surgery may be considered in patients with evidence of myocardial ischemia [68•]. Najm et al. reported unroofing of the intraseptal left coronary artery by circumferentially transecting and extending the right ventricular infundibulum with promising outcomes in 14 patients [83, 84••]. Our center performed supraarterial myotomy of the intraseptal segment through a right ventriculotomy and TAR of the left coronary artery, resulting in improved physiological testing following surgery, and return to competitive wrestling [23]. Although study showed promising improvement in coronary perfusion, further studies are warranted to determine long-term outcomes and refine indications for repair [68•].

## Computational Modeling and Future Direction

The complexity of AAOCA combined with the undefined mechanisms of myocardial ischemia and SCA have motivated a partnership between the clinical team and analytic team to spearhead efforts to construct physics-based computer models to explore hypotheses related to myocardial ischemia in a controlled experimental environment. Generally, computational models can be classified based on their level of spatial detail, ranging from computationally efficient zero-dimensional (0D) models, that do not directly incorporate spatial variations, to high-fidelity three-dimensional (3D) models.

The simplest 0D models can be constructed utilizing imaging data in patients with AAOCA, predicting flow at rest and in hyperemia [85•]. Higher fidelity 3D models have also been used to describe AAOCA physiology and predict flow and pressure fields within the aortic root and coronary arteries [86•]. Time-averaged wall shear stress can be measured and shown to decrease post unroofing surgery in AAOCA [86•]. Several model geometries can also be created for a single patient with varying degrees of narrowing within the intramural segment [87•]. Additionally, other 3D studies neglect the fluid mechanics and instead consider only the vessel wall deformations. Loading simulations can be performed to study the luminal compression of the anomalous coronary artery [88, 89•]. While these studies considered the 3D nature of the aorta and coronary anatomy, they did not directly take into account the two-way coupling of the vessel wall deformations and the blood flow, a key aspect in compression of the intramural segment. Jiang et al. addresses this modeling challenge by creating fluid structure interaction models for adult patients with R-AAOCA [90••]. They demonstrated reasonable agreement between clinically measured and model predicted FFR and iFR in rest and stress conditions. Puelz et al. also considered fluid structure interaction models in pediatric patients with R-AAOCA and L-AAOCA (Supplemental Fig. 6). The focus of this study was on the calibration of downstream boundary conditions using the resting FFR, with the goal of more accurately predicting FFR during stress conditions [91•].

Several challenges remain in computational modeling for AAOCA and its clinical application. Firstly, linking myocardial ischemia with exercise in these patients presents a hurdle, requiring credible models of control mechanisms during physical activity. Although computational approaches for control in exercise exist, they are actively researched, especially in congenital heart disease. Secondly, image-based geometries are important in computational approaches for AAOCA, which are often derived from patient medical images. Challenges include the resolution of the anomalous coronary lumen and the approximation of the intramural segment; both are vital for accurate AAOCA morphology reconstruction and expected to improve with imaging technological advancements. Lastly, the interaction of coronary flow with the aortic valve leaflets is often overlooked. Computer models typically omit any description of the valve because it is challenging to handle from a computational point-of-view and the valve leaflet geometries are not available from medical imaging data that are typically acquired in patients with AAOCA. While challenging computationally, recent methods have enabled the construction of patient-specific valve geometries [92•]. We hope that future computational approaches for AAOCA will address the interactions between moving valve leaflets and anomalous coronary flow.

## Conclusions

AAOCA presents both diagnosis and management challenge across various age groups with a spectrum of symptoms severity from asymptomatic patients to SCA/SCD. Existing consensus guidelines for the diagnosis and management of AAOCA are hindered by a lack of definitive evidence resulting in considerable variability in risk assessment and treatment decisions, particularly for asymptomatic patients. A standardized approach to patient evaluation, incorporating meticulous data collection and multi-center collaboration holds promise for improving risk stratification and guiding optimal management decisions. Both non-invasive assessment and invasive evaluation under provocative stress have emerged as pivotal tools for initial and postoperative evaluation. Surgical techniques tailored to specific AAOCA subtypes have evolved with surgical unroofing and TAR emerging as the most utilized techniques for intramural R-AAOCA and L-AAOCA. Implementing standardized approaches would not only ensure consistent care but also promote a safer environment for individuals with AAOCA to engage in physical activities and competitive sports. Despite facing with challenges, computational modeling holds potential and enables testing of various hypotheses related to myocardial ischemia in AAOCA. Collaborative efforts and further research are vital in refining optimal evaluation and management strategies for AAOCA, aiming to improve outcomes and the quality of life for affected individuals and families.

## Supplementary Information

Below is the link to the electronic supplementary material.

Supplemental Figure 1. Normal coronary anatomy and AAOCA subtypes. Printed with permission from Texas Children’s Hospital. Printed with permission from Texas Children’s Hospital (PDF 57 KB)

Supplemental Figure 2. Evaluation and Management of Anomalous Aortic Origin of a Right Coronary Artery. Printed with permission from Texas Children’s Hospital (PDF 204 KB)

Supplemental Figure 3. Evaluation and Management of Anomalous Aortic Origin of a Left Coronary Artery. Printed with permission from Texas Children’s Hospital (PDF 270 KB)

Supplemental Figure 4. Proposed algorithm to select surgical intervention techniques for patients with AAOCA based on coronary artery anatomy using computerized tomography angiography and surgical inspection.(32) TAR: Transection and Reimplantation (PDF 144 KB)

Supplemental Figure 5. Diagrams of surgical unroofing of an intramural course (A) versus transection and reimplantation (B) based on anatomic features on CTA and surgical inspection. Modified and printed with permission from Texas Children’s Hospital (PDF 205 KB)

Supplemental Figure 6. An example workflow for the construction and simulation of fluid-structure interaction models for AAOCA. (1) Model geometries are based on segmentation of CTA data. (2) 3D reconstruction of the segmented CTA data. (3) Finite element mesh for the vessel wall created from this segmentation. (4) Creation of the aortic valve leaflets and corresponding fibers used in the material model for the leaflet tissue. (5) Completed mesh used in the computer simulations. (6) Streamlines of the blood velocity field from the simulation. Due to the inclusion of the deforming aortic valve leaflets, simulations were executed using a version of the immersed boundary method. (PDF 446 KB)

## Data Availability

The data underlying this article will be shared on reasonable request to the corresponding author.

## Abbreviation

AAOCA: Anomalous aortic origin of a coronary artery
AED: Automated external defibrillator
CAAP: Coronary Artery Anomalies Program
CTA: Computed tomography angiography
DSCMR: Dobutamine stress cardiac magnet resonance imaging
FFR: Fractional flow reserve
iFR: Instantaneous wave-free ratio
IQR: Interquartile range
IVUS: Intravascular ultrasound
L-AAOCA: Anomalous aortic origin of the left coronary artery
NPI: Nuclear perfusion imaging
R-AAOCA: Anomalous aortic origin of the right coronary artery
RWMA: Regional wall motion abnormalities
SCA: Sudden cardiac arrest
SCD: Sudden cardiac death
SPI: Stress perfusion imaging
TAR: Transection and reimplantation
