# Supplementary material for: Anomalous Aortic Origin of a Coronary Artery in Pediatric Patients
Source: Curr Pediatr Rep. 2024 May 24;12(3):69–80. doi: 10.1007/s40124-024-00317-7 (PMC11729077; doi:10.1007/s40124-024-00317-7)
Supplement: Supplementary file 1 — Supplemental Figure 1. Normal coronary anatomy and AAOCA subtypes. Printed with permission from Texas Children’s Hospital. Printed with permission from Texas Children’s Hospital (PDF 57 KB) [file 40124_2024_317_MOESM1_ESM.pdf]

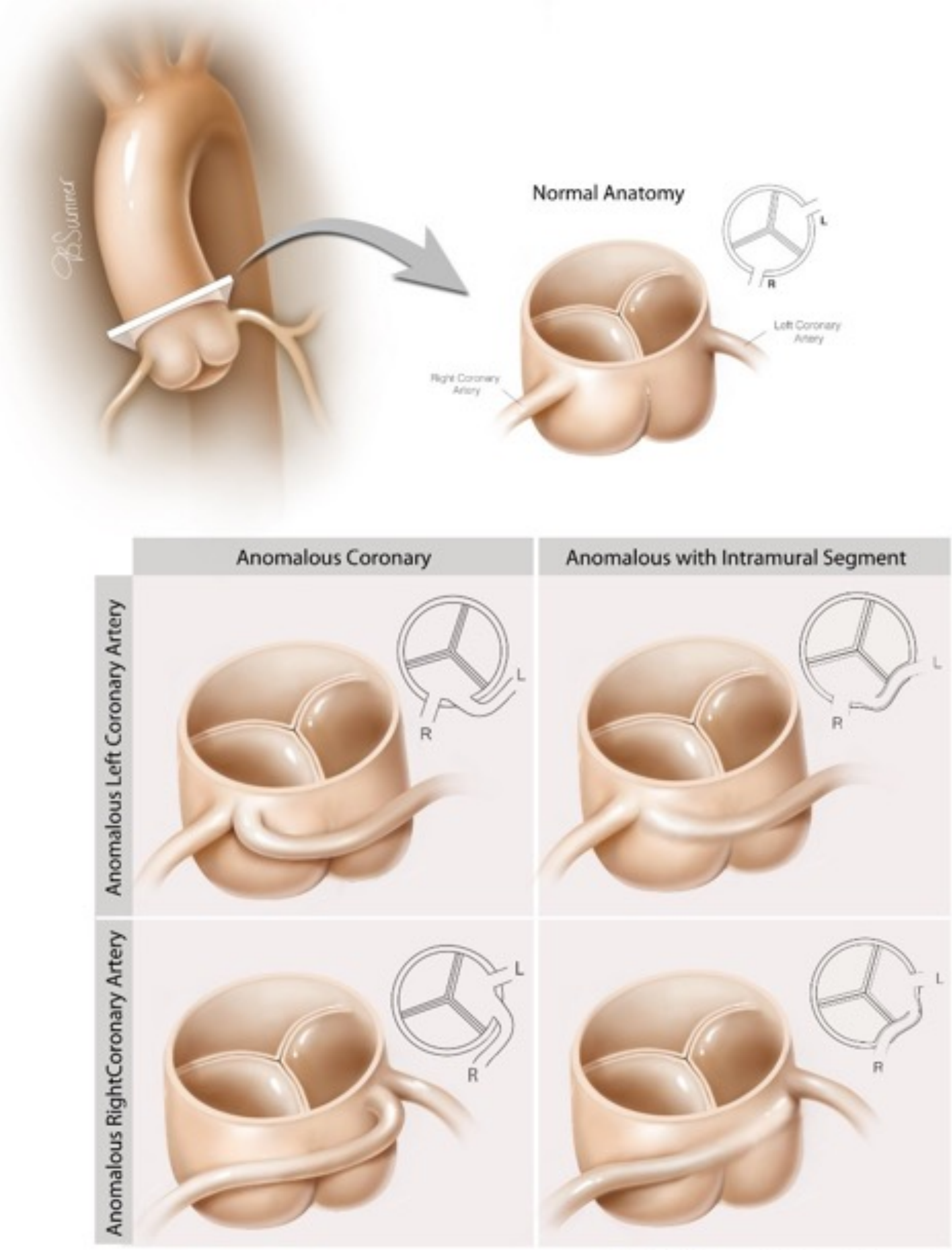

© 2013 Texas Children's Hospital
