# Supplementary material for: Anomalous Aortic Origin of a Coronary Artery in Pediatric Patients
Source: Curr Pediatr Rep. 2024 May 24;12(3):69–80. doi: 10.1007/s40124-024-00317-7 (PMC11729077; doi:10.1007/s40124-024-00317-7)
Supplement: Supplementary file 2 — Supplemental Figure 2. Evaluation and Management of Anomalous Aortic Origin of a Right Coronary Artery. Printed with permission from Texas Children’s Hospital (PDF 204 KB) [file 40124_2024_317_MOESM2_ESM.pdf]

Coronary Artery Anomalies Program

Texas Children's Heart Center

Texas Children's Hospital®

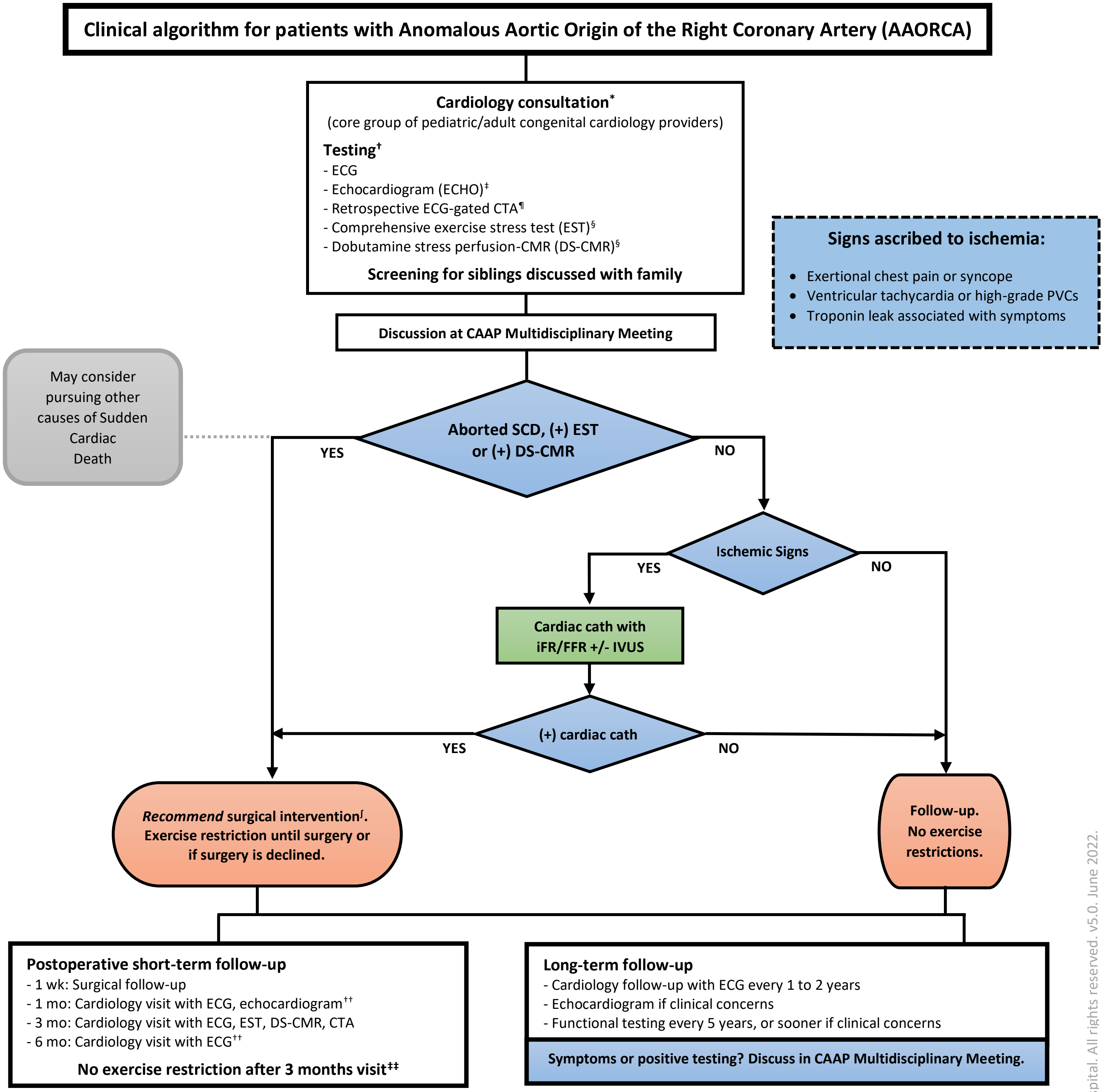

CAAP: Coronary Artery Anomalies Program, DS-CMR: Dobutamine stress cardiac magnetic resonance imaging, EST: Exercise stress test, FFR: fractional flow reserve, iFR: Instantaneous wave-free ration, IVUS: intravascular ultrasound, SCD: Sudden cardiac death.

* Consent obtained for participation in prospective registries.

† Additional studies (i. e. Holter) may be performed depending on the clinical assessment.

‡ External echocardiograms do not need to be repeated if the study is deemed appropriate.

§ EST or DS-CMR may be deferred until approximately 11-12 years of age, unless clinical concerns. DS-CMR and EST deferred if post aborted SCD.

¶ An external CTA may be used if able to upload the images and the study provides all necessary information for management decision-making. CTA should be deferred until approximately 10 years of age, unless clinical concerns.

∫ Aspirin will be administered for 3 months after surgery.

†† Patient may be seen by outside primary cardiologist.

‡‡ Postoperative patients will be cleared for exercise and competitive sports based on findings at the third month postoperative visit including results of CPET, DS-CMR, and CTA (as well as cardiac cath, if done preoperatively).

© 2022 Texas Children's Hospital. All rights reserved. v5.0. June 2022.
