# Supplementary figures and images for: Anomalous Aortic Origin of a Coronary Artery in Pediatric Patients
Source: Curr Pediatr Rep. 2024 May 24;12(3):69–80. doi: 10.1007/s40124-024-00317-7 (PMC11729077; doi:10.1007/s40124-024-00317-7)

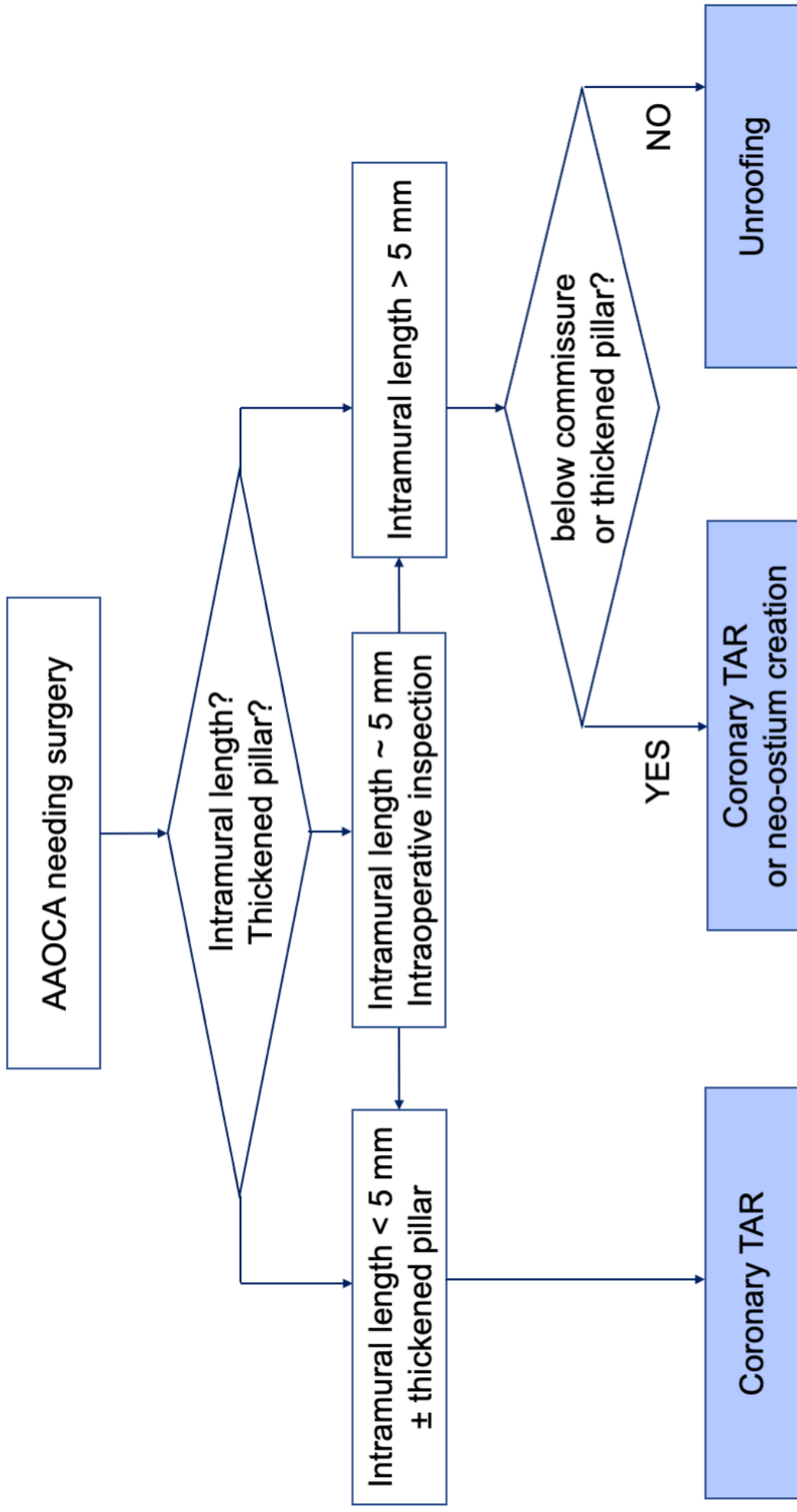

Supplement: Supplementary file 4 — Supplemental Figure 4. Proposed algorithm to select surgical intervention techniques for patients with AAOCA based on coronary artery anatomy using computerized tomography angiography and surgical inspection.(32) TAR: Transection and Reimplantation (PDF 144 KB) [file 40124_2024_317_MOESM4_ESM.pdf]
