# Supplementary material for: Anomalous Aortic Origin of a Coronary Artery in Pediatric Patients
Source: Curr Pediatr Rep. 2024 May 24;12(3):69–80. doi: 10.1007/s40124-024-00317-7 (PMC11729077; doi:10.1007/s40124-024-00317-7)
Supplement: Supplementary file 5 — Supplemental Figure 5. Diagrams of surgical unroofing of an intramural course (A) versus transection and reimplantation (B) based on anatomic features on CTA and surgical inspection. Modified and printed with permission from Texas Children’s Hospital (PDF 205 KB) [file 40124_2024_317_MOESM5_ESM.pdf]

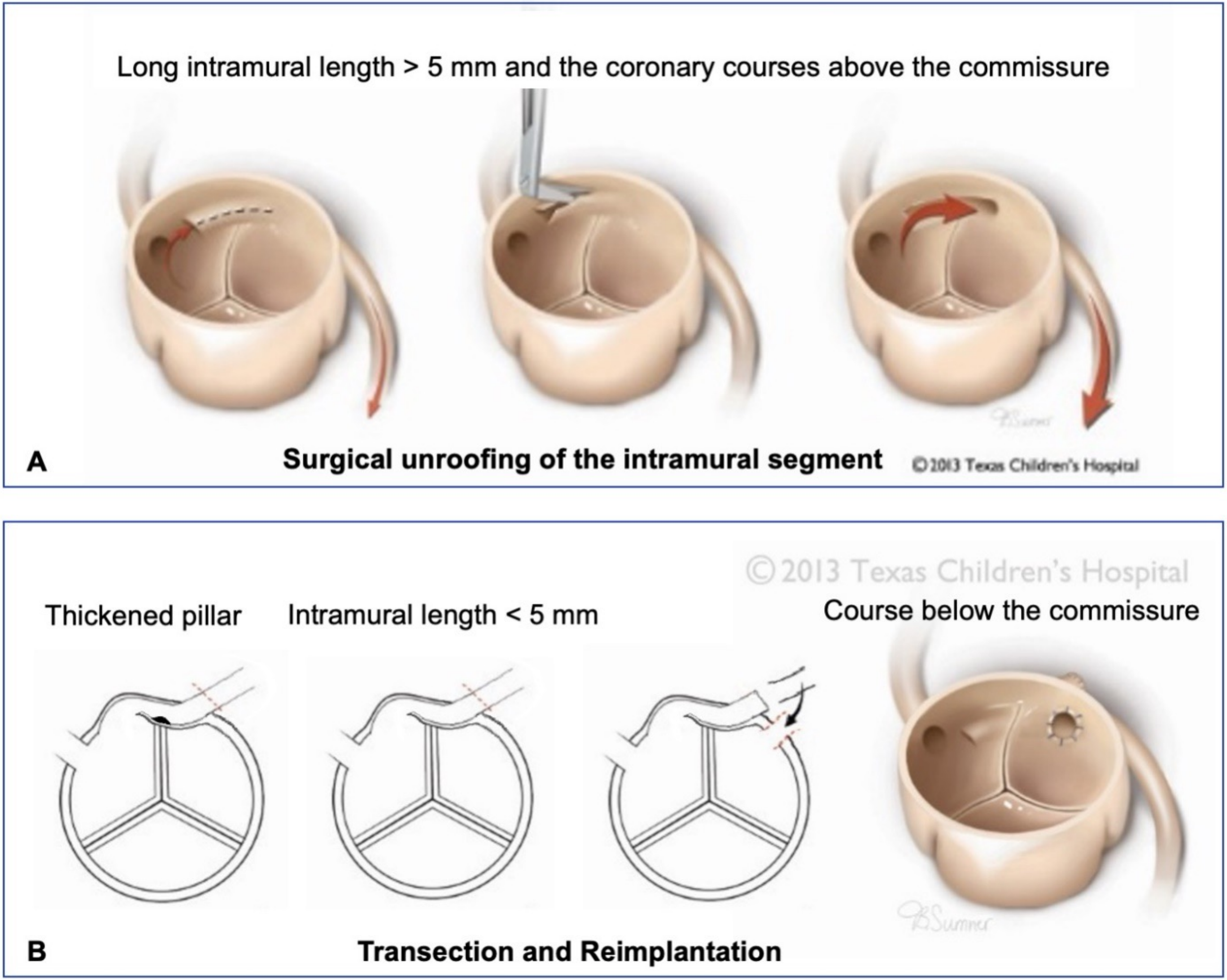
Long intramural length > 5 mm and the coronary courses above the commissure
A
Surgical unroofing of the intramural segment
©2013 Texas Children's Hospital
©2013 Texas Children's Hospital
Thickened pillar
Intramural length < 5 mm
Course below the commissure
B
Transection and Reimplantation
