# Supplementary material for: Anomalous Aortic Origin of a Coronary Artery in Pediatric Patients
Source: Curr Pediatr Rep. 2024 May 24;12(3):69–80. doi: 10.1007/s40124-024-00317-7 (PMC11729077; doi:10.1007/s40124-024-00317-7)

Workflow for 3D Fluid Structure Interaction Models of Aortic and Coronary Flow

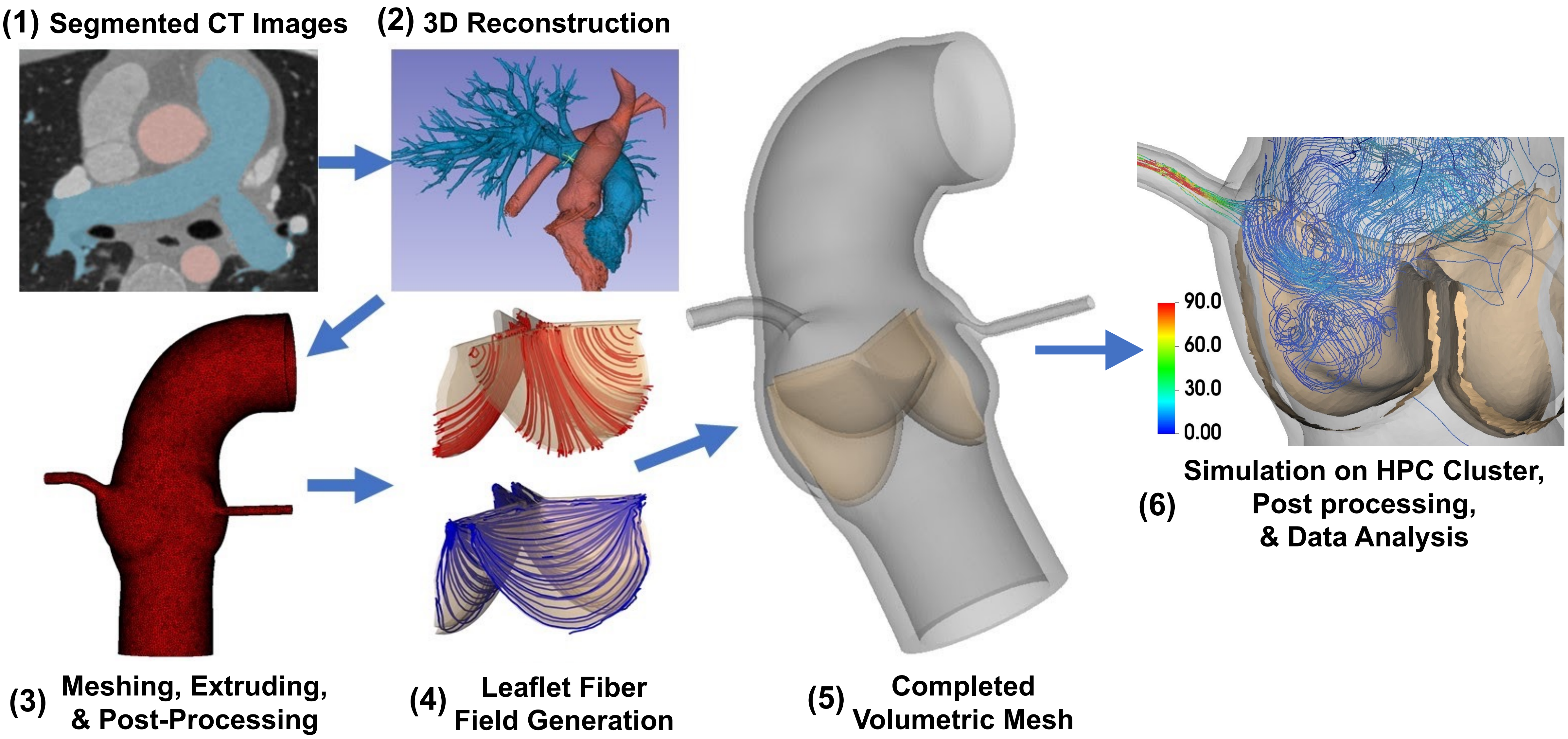

Supplement: Supplementary file 6 — Supplemental Figure 6. An example workflow for the construction and simulation of fluid-structure interaction models for AAOCA. (1) Model geometries are based on segmentation of CTA data. (2) 3D reconstruction of the segmented CTA data. (3) Finite element mesh for the vessel wall created from this segmentation. (4) Creation of the aortic valve leaflets and corresponding fibers used in the material model for the leaflet tissue. (5) Completed mesh used in the computer simulations. (6) Streamlines of the blood velocity field from the simulation. Due to the inclusion of the deforming aortic valve leaflets, simulations were executed using a version of the immersed boundary method. (PDF 446 KB) [file 40124_2024_317_MOESM6_ESM.pdf]
